# Regulatory T cells targeting a pathogenic MHC class II: Insulin peptide epitope postpone spontaneous autoimmune diabetes

**DOI:** 10.3389/fimmu.2023.1207108

**Published:** 2023-08-01

**Authors:** Nyerhovwo Obarorakpor, Deep Patel, Reni Boyarov, Nansalmaa Amarsaikhan, Joseph Ray Cepeda, Doreen Eastes, Sylvia Robertson, Travis Johnson, Kai Yang, Qizhi Tang, Li Zhang

**Affiliations:** ^1^ Diabetes Center, Indiana Biosciences Research Institute, Indianapolis, IN, United States; ^2^ Department of Medicine, Endocrinology, Diabetes & Metabolism, Baylor College of Medicine, Houston, TX, United States; ^3^ Department of Biostatistics and Health Data Science, School of Medicine, Indiana University, Indianapolis, IN, United States; ^4^ Melvin and Bren Simon Comprehensive Cancer Center, Experimental and Developmental Therapeutics, School of Medicine, Indiana University, Indianapolis, IN, United States; ^5^ Center for Computational Biology and Bioinformatics, School of Medicine, Indiana University, Indianapolis, IN, United States; ^6^ Herman B Wells Center for Pediatric Research and Department of Pediatrics, Indiana University School of Medicine, Indianapolis, IN, United States; ^7^ School of Medicine, Indiana University Bloomington, Bloomington, IN, United States; ^8^ Diabetes Center, University of California San Francisco, San Francisco, CA, United States; ^9^ Department of Surgery, University of California San Francisco, San Francisco, CA, United States; ^10^ Gladstone Institute of Genomic Immunology, University of California San Francisco, San Francisco, CA, United States; ^11^ Center for Diabetes and Metabolic Diseases, Indiana University School of Medicine, Indianapolis, IN, United States; ^12^ Biochemistry & Molecular Biology, Indiana University School of Medicine, Indianapolis, IN, United States

**Keywords:** Type 1 diabetes, regulatory T cell, chimeric antigen receptor, antigen specific immunotherapy, MHC II/Insulin complex, monoclonal antibody

## Abstract

**Introduction:**

In spontaneous type 1 diabetes (T1D) non-obese diabetic (NOD) mice, the insulin B chain peptide 9-23 (B:9-23) can bind to the MHC class II molecule (IA^g7^) in register 3 (R3), creating a bimolecular IA^g7^/InsulinB:9-23 register 3 conformational epitope (InsB:R3). Previously, we showed that the InsB:R3-specific chimeric antigen receptor (CAR), constructed using an InsB:R3-monoclonal antibody, could guide CAR-expressing CD8 T cells to migrate to the islets and pancreatic lymph nodes. Regulatory T cells (Tregs) specific for an islet antigen can broadly suppress various pathogenic immune cells in the islets and effectively halt the progression of islet destruction. Therefore, we hypothesized that InsB:R3 specific Tregs would suppress autoimmune reactivity in islets and efficiently protect against T1D.

**Methods:**

To test our hypothesis, we produced InsB:R3-Tregs and tested their disease-protective effects in spontaneous T1D NOD.CD28^-/-^ mice.

**Results:**

InsB:R3-CAR expressing Tregs secrete IL-10 dominated cytokines upon engagement with InsB:R3 antigens. A single infusion of InsB:R3 Tregs delayed the onset of T1D in 95% of treated mice, with 35% maintaining euglycemia for two healthy lifespans, readily home to the relevant target whereas control Tregs did not. Our data demonstrate that Tregs specific for MHC class II: Insulin peptide epitope (MHCII/Insulin) protect mice against T1D more efficiently than polyclonal Tregs lacking islet antigen specificity, suggesting that the MHC II/insulin-specific Treg approach is a promising immune therapy for safely preventing T1D.

## 1 Introduction

Antigen-specific therapy is highly desirable for treating type 1 diabetes (T1D). It has been known for decades that insulin is a key autoantigen for T1D (1). Non-obese diabetic (NOD) mice expressing only insulin with a single mutation in the B-chain amino acid 16 from tyrosine to alanine are completely protected against T1D (2). Insulin B (InsB):9-23 peptides contain key targets of islet autoimmunity, leading to T1D in a spontaneous diabetic NOD mouse model (3–8), which is also likely in humans (9–14). B:9-23 peptides can bind to more than one position or register within the antigen-binding groove of IA^g7^. Weak antigen binding can cause the escape of pathogenic T cells from thymic negative selection, eventually causing autoimmune diseases (15–18). By trapping the peptide in each potential binding register of IA^g7^, Kappler et al. demonstrated that most, if not all, islet infiltrating B:9-23 specific CD4^+^ T cells in NOD mice recognized the insulin B:9-23 peptide presented in register 3 by IA^g7^ (the InsB:R3 epitope) (7, 19). These findings establish that InsB:R3 is a key autoantigen that is critical for initiating diabetogenesis in NOD mice. The fact that the recombinant IA^g7^/InsB:R3 protein vaccine can postpone T1D indicates that the InsB:R3 conformational epitope is a prime target for developing islet antigen-specific immune interventions (20). Given their inherent specificity, islet-antigen-specific immune therapies are expected to dominantly suppress diverse islet autoimmune effector cells in a targeted, tissue-restricted manner; therefore, they are greatly desired for treating T1D (21, 22).

We previously generated two InsB:R3 epitope-specific monoclonal antibodies, mAb287 and mAb757, from the B cell clones of immunized mice. Such antibodies do not bind to free insulin peptides, complexes with insulin peptides bound in other registers, or complexes with those containing non-insulin peptides (23, 24). Administration of such antibodies substantially reduced the severity of insulitis and delayed or prevented the onset of T1D in insulitis-established prediabetic NOD mice, regardless of euglycemia or impaired glucose tolerance (23).

Although effective, the protection obtained via monoclonal antibody therapy relies on weekly injections. We further reported that a single dose of InsB:R3-specific T cell treatment was effective in preventing T1D (25). Genetically engineered T cells with a chimeric antigen receptor (CAR) have a new antigen specificity via a single-chain antibody. Engineered CAR-T cells have been successfully used in cancer treatments (26, 27). We constructed InsB:R3 specific CAR (InsB:R3-CAR) using a single-chain Fab antibody of mAb287. CAR-transduced T cells retained the InsB:R3 specificity of mAb287 (25). Upon engaging with InsB:R3 antigens, the transduced CD8 ^+^ T cells release proinflammatory cytokines and selectively kill antigen-presenting cells carrying the antigens. Adoptively transferred InsB:R3-CAR expressing CD8 T cells selectively home to and survive in islet lesions and pancreatic lymph nodes (PLNs), where relevant islet antigens can be found. A single infusion of InsB:R3 -CD8 T cells is sufficient to eliminate insulin autoantibody-producing B cells and reduce the severity of insulitis (25). The success of InsB:R3-CAR guided T cells in postponing T1D has encouraged us to pursue alternative InsB:R3-targeted immune therapies to treat T1D more efficiently.

Treg therapy is a promising approach for autoimmune diseases, as Tregs maintain immune homeostasis and promote immune tolerance. Specifically, Tregs maintain this immune modulatory role by suppressing proinflammatory effector T cells (Teffs) and other immune cell subsets, directly by cell-cell contact and indirectly by secreting suppressive cytokines (28–31). Furthermore, Tregs are defective in quantity and functionality in T1D patients and NOD mice (32–38). Thus, Treg infusion is an attractive emergent therapy for treating autoimmune diseases to regain the immune balance between pathogenic autoreactive and protective regulatory immune responses (39, 40). To efficiently suppress islet inflammation, Tregs must migrate to pancreatic islets and draining lymph nodes. Islet antigen-specific Tregs readily home to the relevant target tissues (41–44), whereas Tregs lacking islet antigen specificity do not proliferate well in the PLNs (45). The ability of InsB:R3-CAR to guide T cells to inflamed islets and pancreatic lymph nodes suggests that it may also drive Tregs to selectively accumulate in damaged islets and suppress pathogenic T cells. In T1D, beta cells are damaged by islet antigen-reacting and bystander T cells. Given the high specificity of InsB:R3-CAR and the dominant suppressive function of Tregs (capable of controlling effector T cells regardless of their antigen specificity), we hypothesized that InsB:R3-CAR-expressing Tregs could selectively suppress islet inflammatory effector cells, thereby inhibiting and halting T1D progression.

In NOD mice, B:9-23 peptide reactive CD4 T cells can be categorized as either “type A” in response to the core sequence of B13-21 or “type B” preferentially recognizing B12-20 (16, 46, 47). Previously, we found that two B:9-23 mimotopes (B:9-23p8E and B:9-23p8G) enhanced the register 3 binding and acted as potent agonists for “type A” and “type B” T cells (7, 19, 48–50). The original InsB:R3-CAR used in the CD8 CAR-T cell study was constructed with mAb287, which showed low and negligible affinity for the B:9-23p8E and B:9-23p8G mimotopes, respectively. In contrast, mAb757 showed a higher affinity for both mimotopes and provides a more substantial suppression of both types of T cells. Therefore, we built a high-affinity InsB:R3-CAR using single-chain Fab fragments of mAb757 and co-stimulatory domains of second-generation CARs (G2-CARs).

In this study, we generated various InsB:R3-CARs, expressed them in mouse Tregs, characterized their regulatory features, investigated their suppressive function, and determined their disease protective in NOD.CD28^-/-^ mice, a strain of NOD mice with a more aggressive spontaneous T1D (51). The present study demonstrated that Tregs specific for the IA^g7^/InsB:R3 epitope can inhibit the development of T1D more efficiently than polyclonal Tregs lacking islet antigen specificity. Our data suggest that the MHC II/insulin-specific Treg approach can be leveraged to achieve robust protection for future immune therapies.

## 2 Materials and methods

### 2.1 Animals

NOD.CD28^-/-^ mice (41, 51) were kindly provided by Dr. Tang (UCSF, San Francisco, CA, USA). NOD/LtJ, Thy1.1 NOD, FoxP3^GFP^.NOD, and NSG (NOD scid gamma) mice were purchased from Jackson Laboratories (Bar Harbor, ME). All mice were bred and maintained under specific pathogen-free conditions, with 12-hour light/dark cycles and food and water ad libitum, in accordance with protocols approved by the Indiana University Animal Care and Use Committee.

### 2.2 Generation and validation of CAR constructs

Ab757 was used to build high-affinity InsB:R3-CAR constructs following a published protocol (25). **Supplemental Figure 1** illustrates the CAR constructs. The light chain was linked to the V region and CH1 (VH-CH1) domain through a spacer region and fused sequentially to the stalk, transmembrane, and cytoplasmic domains of mouse CD28 and the cytoplasmic domain of mouse CD3ζ (CD247; residues 52-164). The cassettes were cloned into the pMIGII retroviral backbone carrying the blue fluorescent protein (BFP). Ab24.1 (ATCC HB-11947) recognizes an intracellular antigen of human cystic fibrosis transmembrane conductance regulator (CFTCR) (25). Therefore, Ab24.1 was used to generate control CAR (Ctl-CAR). To validate the binding specificity of CARs, we initially expressed them in 5KC TCR^-^ cells that do not express functional endogenous T cell receptor (TCR) (25, 52). Replication-defective retroviruses encoding CARs were generated in Phoenix ECO cells (ATCC CRL-3214). 5KC^-/-^ cells were co-transfected with CAR plasmids and pCL-Ecoplasmids at a ratio of 3:1 using Lipofectamine 3000 (Life Technologies). CAR-BFP^+^ 5KC cells were sorted and stimulated with plate-bound IA^g7^/InsB:R3, IA^g7^/InsB:R3, M12C3, or CFTCR-expressing M12C3 cells. Secreted mouse IL-2 was measured using ELISA to evaluate the specificity and function of the CARs. The specificity and key components of InsB:R3-CARs are summarized in **Table 1**.

**Table 1:** CARs used in InsB:R3-Tregs and Ctl-Tregs.

CAR-Tregs	Parental Ab of the CAR (24, 25)	Epitope and antigen binding affinity (KDs) (24)	Costimulatory domain in CAR (25)
InsB:R3-Tregs	Ab757	5.6x 10^-9^ M to IA^g7^/InsB:R3p8G 1.3 x 10 ^-8^ M to IA^g7^/InsB:R3p8E	CD28, CD3ζ
Ctl-Tregs	Ab24.1	cystic fibrosis transmembrane conductance regulator (CFTCR)	CD28, CD3ζ

### 2.3 Generation and expansion of primary CAR-Tregs

To generate primary CAR-T cells, splenocytes, and lymph node cells of 6–8 weeks old Foxp3^GFP^ NOD mice were isolated on day 0. CD4^+^CD25^+^ T cells were enriched using a CD4^+^CD25^+^ Regulatory T Cell Isolation Kit (Miltenyi Biotec), and live Tregs gated on DAPI^-^ CD8^-^ CD4^+^CD25^hi^ CD62L^+^ FoxP3 GFP^+^ were sorted. The sorted Tregs were activated *in vitro* using Dynabeads coated with mouse CD3/CD28 antibodies at a 3:1 ratio of beads to cells for 48 h in DMEM containing 10% heat-inactivated fetal bovine serum and human recombinant IL-2 (1000 IU/ml; PeproTech). Retroviruses encoding the CARs were freshly produced in Phoenix ECO cells. On day 2, the activated Tregs were transferred to retronectin (Clontech)-coated 48-well plates at a density of 0.5x10^6^/well, and approximately 1.2 ml filtered retrovirus particles were added. IL-2 was added to the transduction wells to attain a final concentration of 1000 U/ml, and the cells were spin transduced at 2000g for 90 min at 37°C. The second transduction was performed on the following day. The transduced cells were washed and expanded in a medium containing IL-2 and Dynabeads. CAR-expressing Tregs were sorted on day 8-9 gated on a live CD4^+^CD25^hi^CAR^BFP+^Foxp3^GFP+^ population (**Supplemental Figure 2**). The purity of the sorted CARTregs was above 90%. The sorted CAR-Tregs were either transferred to mice immediately or restimulated with CD3/CD28 beads at a 1:1 ratio of beads to cells for four days before being transferred. In each experiment, a portion of the transduced Tregs were used to test the expression of Foxp3 routinely. More than 95% of Tregs expressed Foxp3.

### 2.4 MHCII/peptide tetramer and Treg biomarker staining

The binding specificity of the CAR-Tregs was confirmed using IA^g7^/Insulin tetramers and control tetramers (provided by the National Institute of Health Tetramer Core Facility at Emory University). Following Fc blocking, CAR- Tregs (2 × 10^5^) were stained with IA^g7^/Insulin tetramer (p8E and p8G cocktail) or IA^g7^/HEL control tetramer (Hen Egg Lysozyme) at 37°C for 75 min, then anti-mouse CD4 and anti-mouse CD25 were added and incubated for 15 min at 37°C. For Foxp3 staining, cells were stained with anti-CD4 and anti-mouse CD25 at 4°C for 20 min, fixed and permeabilized following the manufacturer’s instructions (Miltenyi Biotec 130-093-142), and stained with REAfinity anti-mouse FoxP3-APC (Miltenyi Biotec 130-111-679). After washing, the samples were run on a Cytek Aurora and the data were analyzed using FlowJo software (Tree Star). All other antibodies were purchased from eBioscience Inc. or BioLegend Inc.

### 2.5 Antigen-specific proliferation and activation assays of primary CAR-Treg cells

The activation of CAR-Tregs was evaluated by measuring cytokine secretion in response to CAR ligands. Previously, the insulin B:9-23 peptide with amino acid 22 R mutated to E was fused to the N-terminus of the beta chain of IA^g7^ and expressed in the M12C3 B cell line (53). Therefore, M12C3 B cell lines expressing surface IA^g7^/InsB:R3 or IA^g7^/HEL were irradiated and used as ligands and negative stimuli for the InsB:R3-CAR Tregs. Forty thousand primary CAR-Tregs were cultured with the same number of M12C3 B cells in a round bottom 96-well plates for 16 h in a 10% CO_2_ incubator. The supernatants were collected and the secreted cytokines were measured using the V-PLEX Proinflammatory Panel 1 Mouse Kit (Isoplexis, K15048D-1).

To measure antigen-stimulated proliferation, InsB:R3-Tregs and Ctl-Tregs were labeled with CellTrace Far Red dye (Invitrogen C34564) and plated at 3 × 10^5^/well in 96-well plates. Cells were cultured with the same number of irradiated IA^g7^/InsB:R3- or IA^g7^/HEL- expressing M12C3 cells (3000 Rad) or CD3/CD28 Dyna beads (1 cell:2 beads) in 48-well plates for 4 days. The cells were then stained with CD4 and DAPI Viability Dye, and the dilutions of CellTrace Far Red for CD4^+^CAR^BFP+^Tregs were analyzed using FlowJo proliferation modeling.

To measure the capacity of InsB:R3-Tregs to suppress the proliferation of effector T cells, diabetic splenocytes from Thy1.1 NOD mice were labeled with CellTrace Far Red, plated in round-bottom 96-well plates at a density of 200,000 cells/well, and stimulated with soluble mouse CD3 antibodies at 5 µg/ml. InsB:R3-Tregs were added to the culture at Treg : Teff ratios of 1:5 and 1:10. After 4 days of co-culture, the dilution of CellTrace dye of CD4 and CD8 T cells gated on Thy1.1^+^ splenocytes and CD69 expression were analyzed using flow cytometry.

### 2.6 Survival and distribution of InsB:R3-CAR-Tregs *in vivo*

To analyze the survival and distribution of CAR-Tregs, 2 x10^6^ CAR-Tregs cells generated from Thy1.1 Foxp3^GFP^ donors were transferred to 7-week-old NOD.CD28^-/-^ mice. On day 8 post-transfer, splenocytes, pancreatic lymph node (PLN) cells, and inguinal lymph nodes were collected and stained with antibodies against Thy1.1 and CD4. The frequency of the transferred CAR-T cells was analyzed using flow cytometry. Three mice per group were used for RNA sequence analysis, and two mice per group were used for islet infiltrating lymphocyte flow cytometry.

### 2.7 Islet RNA sequence analysis

The pancreas of mice treated with InsB:R3-Tregs, Ctl-Tregs, or saline buffer (Non-T), three mice per group, was digested, and islets were handpicked on day 8 post-transfer. Using independent parametric significance testing between two groups and assuming a large effect size (Cohen’s d) equal to 4, equal variances, and power of 0.80, significant results (can be achieved with three samples per mouse. Three diabetic NOD.CD28^-/-^ mice (DM) diagnosed within one week were included for comparison. Fresh islets from each mouse were pooled, and RNAs were extracted using an RNA Miniprep kit (Zymo Research, R1054). High-quality RNA samples that passed the quality control test were subjected to RNA-Seq analysis (Novogene Corporation, Inc.).

The proprietary analysis pipeline from Novogene was used for RNA-seq analysis, which is briefly described here. The extracted RNAs was sequenced using Illumina sequencers, resulting in paired-end reads in FASTQ format. High quality reads were obtained by removing reads with adapter sequences, polyN reads > 10%, and reads with Qscore <5 in 50% of bases. These high-quality reads were aligned to the mm10 mouse reference genome using hisat2 (v2.0.5). Read counts were quantified for each gene using featureCounts (v1.5.0-p3). These reads were then converted to fragments per kilobase of transcript per million mapped reads (FPKM) for visualization and dimensionality reduction. The Pearson correlation coefficient (PCC) was calculated for each sample based on FPKM values across the gene set. This PCC matrix was then used as input to principal component analysis (PCA) using the prcomp function with scaling.

Differential gene expression (DGE) analysis was performed on read counts using DESeq2 (v1.20.0), as was described in the DESeq2 user guide. Multiple testing correction was applied to the resulting p-values from the DESeq2 DGE analysis using the Benjamini-Hochberg (BH) correction, resulting in adjusted p-values. Genes with an adjusted p-value 0.05 between conditions were considered differentially expressed genes. After differentially expressed genes (DEGs) were identified, functional enrichment was performed using the clusterProfiler R package in the Gene Ontology (GO) Biological Process, GO Molecular Function, GO Cellular Component, Kyoto Encyclopedia of Genes and Genomes (KEGG), Reactome, Disease Ontology (DO), and DisGeNET databases. Terms from these databases were considered enriched at a BH adjusted p-value 0.05 for each DEG list. All data manipulation, analyses, and visualization after read alignment and quantification were performed in R. Data visualization was primarily performed using the packages described above or with custom ggplot2 functions.

### 2.8 Adoptive transfer of CAR-Tregs for disease prevention

The sorted and expanded G2-InsB:R3-Tregs (0.5, 1 or 2 × 10^6^/mouse) or Ctl-Tregs (2 × 10^6^/mouse) in 100 µl saline were transferred via the tail vein to 5-7 week old NOD.CD28^-/-^ mice. Both male and female mice were used because both sexes were equally likely to develop diabetes. Beginning at 9 weeks, blood glucose levels were monitored weekly using a OneTouch Ultra2 monitor (LifeScan, Inc.). Animals with values of ≥250 mg/dL were re-tested the following day. Diabetes was diagnosed after two consecutive blood glucose values ≥250 mg/dL (23). The mice were euthanized either after the diagnosis of diabetes or at 20 weeks of age. At the end of the experiment, the pancreata and salivary glands were harvested for hematoxylin and eosin staining to assess insulitis and sialitis, respectively. Insulitis was scored blindly by two pathologists according to the following criteria: score 0, intact islets; score 1, peri-insulitis, lymphocyte infiltration restricted to the periphery of the islets; score 2, less than 50% of the islet area is infiltrated; score 3, severe insulitis, 50% or more of the islet area is infiltrated, and the islet structure disrupted.

### 2.9 InsB:R3-Tregs and NOD diabetic splenocytes co-transfer experiment

We collected spleens and lymph nodes from newly diagnosed (within 5 days) diabetic NOD female mice and transferred 10 million splenocytes alone (n=5) or co-transferred them with 2 million InsB:R3-Tregs (n=5) to NSG mice. The blood glucose levels were monitored to determine the incidence of diabetes. Mice were followed up until 40 days post-transfer or until T1D was diagnosed.

### 2.10 Statistical analysis

Survival curves were analyzed using PRISM9 software (GraphPad, San Diego, CA), and *p*-values ≤0.05 were considered statistically significant. The log-rank Mantel–Cox test was used to analyze the time until diabetes development. The differences in immune subpopulations, islet numbers, and insulitis scores between the two groups were analyzed using Student’s t-test. Statistical analyses were performed for RNA-seq data based on the user guide for DESeq2 and the cluster Profiler packages. For visualizing gene expression and direct comparisons without using the R package, FPKM values were used to correct for library size and gene length. Power analysis, as described previously, was used to ensure that a sample size of three per group was sufficient to detect large effect size changes between treatment groups. Multiple testing corrections using the BH method were applied for the DGE analysis and functional enrichment.

## 3 Results

### 3.1 Validation of the InsB:R3-CARs constructed with Ab757

**Figure 1:**
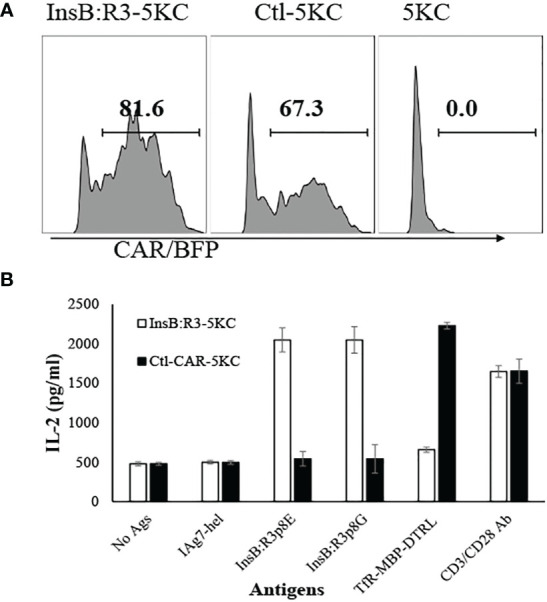
Validation of CARs using 5KC thymomas. **(A)** 5KC cells were transduced with retroviruses particles encoding InsB:R3-CARs and Ctl-CARs, as described in section 2.3. The transduced 5KC cells (2x10^5^/sample) were analyzed by flow cytometry. BFP-positive cells are defined as CAR-expressing cells. **(B)** IL-2 secretion of the 5KC transductants. The InsB:R3-5KCs (blank boxes) and Ctl-5KCs (black boxes) transductants were plated in 96-well plate (10^5^ cells/well) and co-cultured with an equal number of the M12C3 B cell lines expressing antigens as listed overnight in triplicate wells. The secreted IL-2 was measured using ELISA. Means and SD from one representative experiment are shown.

Ab757, having high binding affinities to both p8G and p8E isoforms of IA^g7^/InsB:R3 epitopes (KD 5.6 × 10^-9^ and 1.3 × 10 ^-8^) (24), was used to construct the high-affinity G2 InsB:R3-CAR, as was previously reported. To validate the binding specificity of the CARs, we initially expressed InsB:R3-CAR and Ctl-CAR in 5KC^-/-^ cells, a murine thymoma that lacks expression of endogenous TCRα or β chains (25, 52). As shown in **Figure 1A**, more than 80% of the 5KC cells expressed InsB:R3-CAR-BFP after transduction. Importantly, G2-InsB:R3-CAR expressing 5KC cells secreted robust mouse IL-2 in response to IA^g7^-B:R3 p8E and p8G antigens, but not the control IA^g7^/HEL complexes presented by M12C3 B cell lines (**Figure 1B**). In contrast, 24.1-CAR expressing 5KC cells did not respond to IA^g7^-B:R3 epitopes, but responded robustly to M12C3 cells expressing the TfR-MBP-DTRL fusion protein (data not shown). Our data indicate that InsB:R3-CARs maintain the binding specificity of its parental antibody-Ab75 and are functional in inducing intracellular signals following engagement with their cognate antigens.

### 3.2 InsB:R3-CAR-Tregs maintain the specificity of Ab757 and features of Tregs

**Figure 2:**
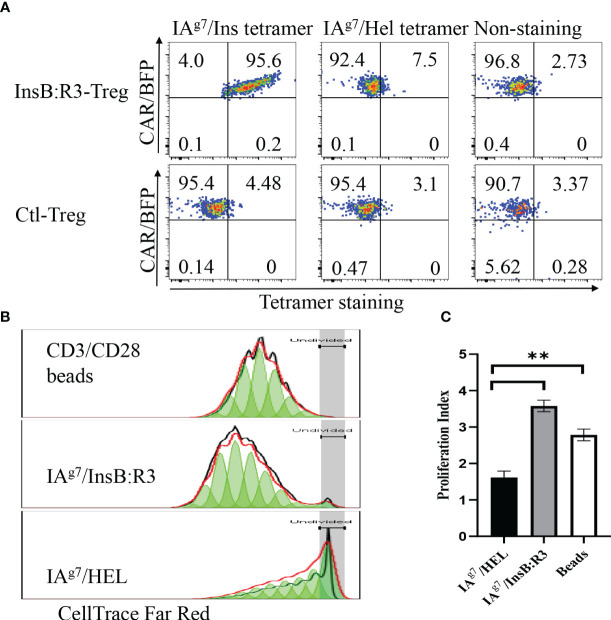
Antigen specificity of InsB:R3-Tregs. **(A)** InsB:R3-CARs expression was measured using IA^g7^-insulin tetramer staining. Two hundred thousand sorted transduced InsB:R3-Tregs or Ctl-Tregs were stained with PE-streptavidin conjugated IA^g7^/Insulin tetramer (left), control IA^g7^/HEL tetramer (hen egg lysozyme, middle), or left unstained (right). DAPI negative cells were gated for tetramer analysis. The percentages are shown in each quadrant. **(B)** Antigen-dependent proliferation of InsB:R3-Tregs. Expanded InsB:R3-Tregs were labeled with CellTrace Far Red and stimulated with CD3/CD28 Dynabeads (1 cell:2 beads), antigens of IA^g7^/InsB:R3, and the control IA^g7^/HEL complexes presented by M12C3 B cell lines for 4 days. Two hundred thousand Tregs per well were plated in 96-well plate. The dilution of CellTrace Far Red was analyzed using FlowJo proliferation model. One representative experiment was shown. **(C)** Proliferation Indices of InsB:R3-Tregs. Bars show average proliferation indices from three independent experiments. Experiments were done in triplicate wells and repeated thrice. ** *p*<0.001 analyzed using Student’s t test.

To characterize the antigen specificities of InsB:R3-Tregs, we transduced cells with retroviral particles encoding InsB:R3-CARs or Ctl-CARs and stained cells with IA^g7^/Insulin tetramers and control IA^g7^/HEL tetramers. The antigen specificities of sorted and expanded CAR-Tregs were analyzed using tetramer staining. As is shown in **Figure 2A**, more than 90% of the sorted CAR-Tregs expressed CARs determined using BFP expression. InsB:R3-Tregs stained positive for IA^g7^/Insulin tetramers (p8E and p8G tetramers) but negative for the IA^g7^/HEL control tetramer. In contrast, Ctl-Tregs did not recognize either the IA^g7^/InsB:R3 tetramer or the IA^g7^/HEL tetramer. To further evaluate the InsB:R3-specific activation and propagation of primary CAR-Tregs, Tregs were labeled with CellTrace Dye and stimulated with IA^g7^/InsB:R3 or IA^g7^/HEL antigens expressed on the M12C3 surface or CD3/CD28 antibody-coated Dynabeads (1 cell:2 beads) as positive controls. After four days of co-culture, InsB:R3-Tregs proliferated in response to CD3/CD28 beads and IA^g7^/InsB:R3 antigens, with mean proliferation indices of 2.8 and 3.6 (**Figures 2B, C**). In contrast, InsB:R3-Tregs did not proliferate in response to the IA^g7^/HEL complexes, with a mean proliferation index of 1.6. Therefore, InsB:R3-CAR is specific to IA^g7^/InsB:R3 antigens and the engagement of CARs with their cognate antigens in the context of MHC II molecules primes CAR-Tregs.

**Figure 3:**
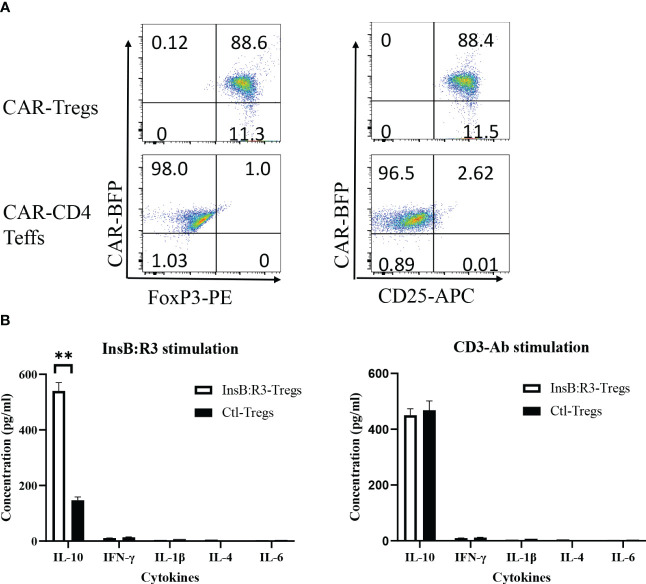
Regulatory characteristics of InsB:R3-Tregs. **(A)** Expanded InsB:R3-Tregs retain FoxP3 expression. The sorted InsB:R3-Tregs were restimulated with CD3/CD28 Dynabeads (1 cell:2 beads) for three days, and cells were stained with CD4 and CD25 antibodies followed with FoxP3 intracellular staining and evaluated via flowcytometry analysis. Cells were gated on CD4 positive cells. **(B)** Cytokine profiles of InsB:R3-Tregs. InsB:R3-Tregs (blank bars) and Ctl-Tregs (black bars) were stimulated with M12C3 cells expressing IA^g7^/InsB:R3 in the medium without IL-2. Following overnight incubation, supernatants were collected, and cytokines were measured as described in section 2.5. Mean values of three independent experiments ± SEM were plotted. *P* values were determined by Student’s t test, ** *p* < 0.001.

To determine whether the expanded CAR-Tregs maintained the features of Tregs, we measured the expression of Foxp3 and determined their cytokine secretion. First, we transduced conventional CD4^+^CD25^-^ T cells (Tcons) and Tregs with InsB:R3-CARs and stained both populations with FoxP3 antibodies. As is shown in **Figure 3A**, approximately 99% of expanded CAR-Tregs expressed FoxP3 and CD25, while no Foxp3 was detected in the InsB:R3-Tcons. Second, we defined InsB:R3-CAR-mediated Treg activation by measuring the secreted cytokines. InsB:R3-Tregs were stimulated with IA^g7^/InsB:R3 or IA^g7^/HEL antigens expressed on the B cell surface. Compared to IA^g7^/HEL, the engagement of IA^g7^/InsB:R3 induced InsB:R3-CAR-Tregs to secrete a robust level of anti-inflammatory cytokines, including IL-10, but neglectable inflammatory cytokines such as IFN γ and IL-1β (**Figure 3B**). This result indicates that stimulation via InsB:R3-CAR in Tregs replicates endogenous TCR signaling, and the expanded InsB:R3-Tregs retain the features of Tregs. Therefore, our data demonstrated that expanded primary InsB:R3-Tregs maintain the regulatory function of Tregs.

### 3.3 Adoptive transfer of InsB:R3-CAR-Tregs significantly delayed the onset of T1D

**Figure 4:**
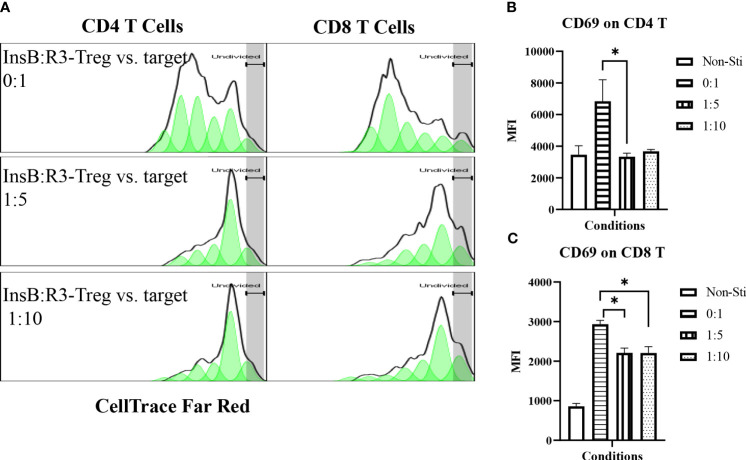
InsB:R3-Tregs inhibit the proliferation of co-cultured polyclonal effector T cells. **(A)** InsB:R3-Tregs suppress the proliferation of CD4 and CD8 T cells. CellTrace Far Red labeled diabetic Thy1.1NOD splenocytes (2x10^5^/well in 96-well plate) were stimulated with mouse CD3 antibodies (5 µg/ml) and cultured alone (InsB:R3-Treg vs. Target as 0:1) or co-cultured with InsB:R3-Tregs at 1:5 and 1:10 ratios of Treg to target. Splenocytes without stimulation (Non-Sti) were used as baseline control. On day 4, cells were stained with antibodies for Thy1.1, CD8, CD4, and CD69 for flow cytometry analysis. The dilution of CellTrace Dye was analyzed as described in section 2.5. Cells were gated on single alive Thy1.1^+^ positive cells. One representative experiment is shown. **(B, C)** InsB:R3-Tregs suppress CD4 and CD8 T cells expressing CD69. Mean MFIs (Median Fluorescence Intensity) of three independent experiments are shown. * *p*<0.05 by Student’s t test.

Tregs suppress effector T cells regardless of their antigen specificities and antigen presenting cells (54). We tested the ability of expanded InsB:R3-CAR-Tregs to suppress the proliferation of polyclonal effector T cells in diabetic NOD mice in response to soluble CD3 antibodies. Diabetic splenocytes from Thy1.1NOD mice were labeled with CellTrace dye and stimulated with soluble CD3 antibodies in the absence or presence of InsB:R3-Tregs (**Figure 4A**). After four days of stimulation, 60.8% of CD4 T cells proliferated for more than one generation. However, only 22% and 23.7% CD4 T cells proliferated more than one generation when co-cultured at Treg: target ratios of 1:5 and 1:10. Approximately 75% of CD8 T cells propagate more than one generation when cultured alone. Co-cultured with InsB:R3-Tregs, only 14% of CD8 T cells proliferated for more than one generation. To evaluate the activation of effector T cells, we measured the expression of CD69, a T cell activation marker. Activated by CD3 antibodies, both CD4 and CD8 T cells expressed relatively high levels of CD69 compared to cells co-cultured with InsB:R3-Treg. **Figures 4B, C** show that InsB:R3-Tregs decreased the mean fluorescence intensity (MFIs) of CD69 in both CD4 ^+^ and CD8 ^+^ T cells. In summary, our data indicated that InsB:R3-Tregs suppress the proliferation and activation of inflammatory polyclonal effector T cells.

Given the suppressive function of InsB:R3-Tregs *in vitro*, we examined whether these cells could delay or prevent T1D in a mouse model of spontaneous autoimmune diabetes, NOD. CD28^-/-^ strain (55, 56). Nearly 100% NOD.CD28^-/-^ mice developed diabetes by week 12, whereas approximately 85% wild type NOD mice developed T1D by week 35. NOD.CD28^-/-^ mice produced the same levels of proinflammatory cytokines in response to islet antigens as wild-type NOD mice. Tregs are defective in quantity and quality in T1D patients and mice (32, 34–37, 55). NOD.CD28^-/-^ mice have a reduced Treg population compared to wild-type NOD mice and have been used to evaluate the function of Tregs (41, 55–57).

**Figure 5:**
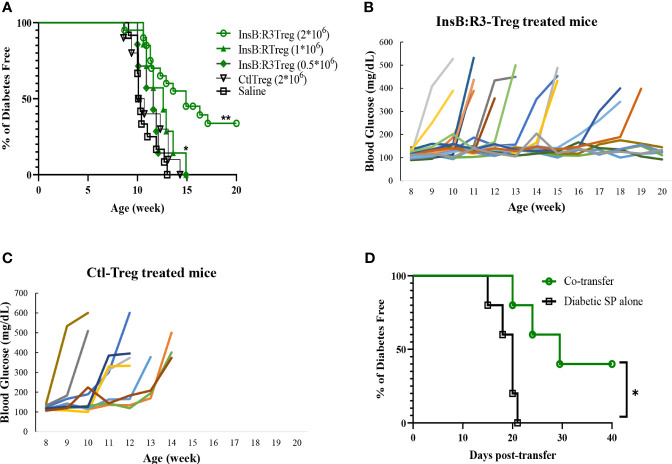
InsB:R3-Tregs inhibit T1D development. **(A)** Groups of 5-7 week old NOD.CD28^-/-^ mice were treated with saline buffer (black squares; n = 12, 5 male, 7 female.), half a million (green diamond; n=7, 3 male, 4 female), 1 million (green triangle; n=7, 3 male, 4 female), or 2 million G2-InsB:R3-Tregs (green circles; n=20, 9 male, 11 female), or 2 million Ctl-Tregs (black inverted triangle; n=10, 4 male, 6 female), and followed up to 20 weeks or until diabetes was diagnosed. Diabetes was diagnosed as described in Materials and Methods. The percentages of remaining diabetes free mice were shown. *P* values were determined using Mantel-Cox test. *p < 0.05 v.s. saline group. ** *p*<0.001 v.s. saline group and *p*=0.001 v.s. Ctl-Treg group. **(B)** Weekly random non-fasting blood glucose levels of individual mice treated with InsB:R3-Tregs or **(C)** control-Tregs are shown. **(D)** InsB:R3-Tregs inhibit diabetic wild type NOD splenocytes (SP) transfer diabetes. NSG mice were transferred with 10x10^6^ NOD splenocytes only (n=5, female) or co-transferred with 2x10^6^ InsB:R3-Tregs (n=5, female), and followed up to 40 days post-transfer or until diabetes was confirmed. The percentages of remaining diabetes free mice of each group are shown. *p < 0.05 determined by Mantel-Cox test.

Five-to seven-week-old prediabetic NOD.CD28^-/-^ mice were treated with InsB:R3-Tregs (0.5, 1, or 2 million), Ctl-Tregs (2 million), or saline buffer. Ctl-Treg and saline groups mice started developing diabetes at the age of 9 weeks, and 100% of the mice, both male and female, developed T1D by 13 weeks (**Figure 5A**). Half a million InsB:R3-Tregs could not slow the progression of T1D. One million InsB:R3Tregs significantly delayed disease development compared with that in the saline group (*p*=0.03), but not in the Ctl-Treg group (*p*=0.28). Two million InsB:R3-Tregs significantly protected mice against the disease compared to the saline (*p*<0.0001) and Ctl-Treg (*p*=0.001) groups. At 11 weeks, 19 of 20 InsB:R3-Treg treated mice remained disease-free, while 40% Ctl-Treg treated mice (2/10) and 58% saline group mice (7/12) already developed disease (both *p*<0.01, Fisher’s exact p test). At 14 weeks, 12 of the 20 InsB:R3-Treg treated mice remained diabetes-free, whereas all saline group mice (12/12) and Ctl-Treg-treated mice (10/10) developed diabetes. The dose-dependent disease protection of InsB:R3-Tregs indicates that a sufficient number of cells or a relatively high ratio of Tregs to Teffs is necessary to suppress disease effectively. The protection of InsB:R3-Tregs remained significant at 20 weeks, a timepoint of two healthy lifespans, with 35% of treated mice maintaining euglycemia. **Figures 5B, C** show the blood glucose levels of the pooled InsB:R3-Treg treated mice, and the Ctl-Treg-treated mice that developed hyperglycemia aggressively. Given the significant protection provided by two million InsB:R3-Tregs, we did not test a higher number of InsB:R3-Tregs. In summary, InsB:R3-Tregs carrying InsB:R3-CARs significantly inhibited the onset of T1D in a dose-dependent manner, whereas Ctl-Tregs lacking islet antigen specificity did not.

We conducted a pilot study to investigate whether InsB:R3-Tregs could suppress wild-type NOD effector cells induced by T1D. Ten million diabetic splenocytes were either transferred alone (n=5) or co-transferred into NSG mice with 2 million InsB: R3-Tregs (n = 5). As expected, all the mice injected with diabetic splenocytes developed diabetes within three weeks (**Figure 5D**). However, the onset of T1D was postponed in the co-transferred mice (*p*<0.05). Forty days post-transfer, two of the five mice remained diabetes-free. Our data indicated that InsB:R3-Tregs can suppress T1D induced by diabetic NOD spleen cells.

### 3.4 InsB:R3-Tregs are relatively enriched at pancreas draining lymph nodes

**Figure 6:**
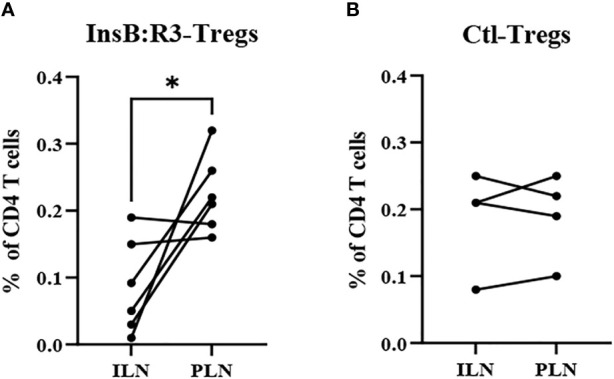
**(A)** InsB:R3-Tregs (n=6) and **(B)** Ctl-Tregs (n=4) in pancreatic draining lymph nodes (PLNs) and control inguinal lymph nodes (ILNs) on day 8 post- transfer. Cells were gated on alive Thy1.1 CD4 T cells. Each dot represents one mouse. Six InsB:R3-Tregs treated mice and four Ctl-Tregs treated mice were plotted. The data between PLNs and ILNs were analyzed with paired Student’s t test, *p<0.05.

The survival of T-cells relies on the engagement of their cognate antigens. Antigen-presenting cells present antigens that activate T cells in the pancreatic lymph nodes (PLNs). Therefore, we generated CAR-Tregs using Thy1.1FoxP3^GFP^ donor mice, treated six NOD.CD28^-/-^ mice with InsB:R3-Tregs and four mice with Ctl-Tregs, and determined the distribution of cells in the PLNs and nondraining inguinal lymph nodes (ILNs). After eight days, the frequency of CAR-Tregs was evaluated using flow cytometry. Compared to ILNs, InsB:R3-Tregs were relatively enriched in pancreatic lymph nodes (*p*<0.05). **Figure 6A** shows that four of six InsB:R3-Treg treated mice had higher frequencies of CAR-Tregs in PLNs than in ILNs (*p* =0.037, two-tailed paired Student’s t-test). However, the Ctl-Treg frequency did not increase significantly between PLNs and ILNs (**Figure 6B**). The enrichment of InsB:R3-Tregs in PLNs was consistent with the specificity of their CARs, supporting their organ-specific distribution. Only four of six InsB:R3-Treg treated mice showed relative enrichment at PLNs, while the other two did not. As only partial disease protection was observed following InsB: R3-Treg treatment (**Figure 5A**), the variability in the enrichment of InsB:R3-Tregs in the PLNs may be associated with their partial disease protection. In a preliminary experiment, the transferred Thy1.1^+^ CARTregs, less than 1% of CD4 T cells, were detected in islets at this time point (**Supplemental Figure 3**). Additionally, the frequency of Tregs in the spleen did not differ between the two groups (data not shown). Therefore, our data suggest that InsB:R3-Tregs home the pancreas-draining lymph nodes and islets in type 1 diabetes.

### 3.5 InsB:R3-Tregs influence immune cell markers and activation related genes in islets

**Figure 7:**
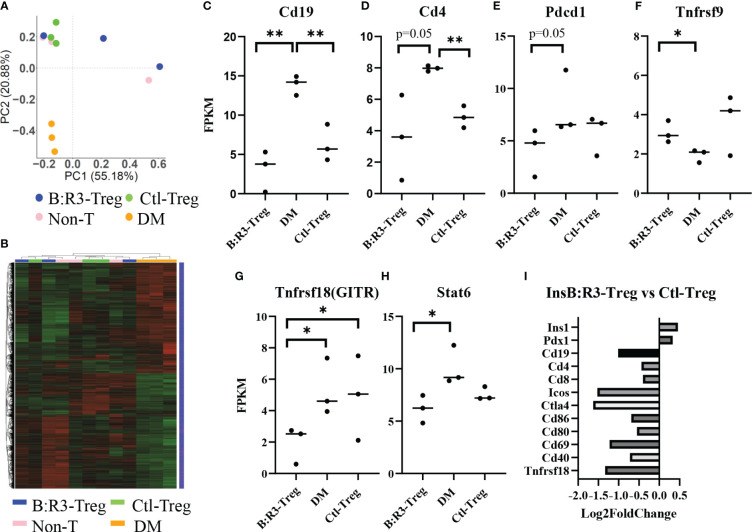
**(A)** PCA on the PCC matrix of gene expression values (FPKM) of all samples. B:R3-Treg (InsB:R3-Treg group). **(B)** The differential expression gene clustering heatmap. Overall results of FPKM cluster analysis, clustered using the log2(FPKM+1) value. Red and green colors indicate genes with high and low expression levels, respectively. The color ranging from red to green indicates that log2(FPKM+1) values from large to small. **(C–H)** Gene expression level estimated by FPKM. *p<0.05, **p<0.01. **(I)** Gene expression changes in InsB:R3-Treg group and Ctl-Treg group. Numbers of the Log2 fold change were plotted.

To analyze the short-term effects of InsB:R3-Tregs on islet inflammation, we extracted islet RNA from individual mice on day 8 post-transfer, as described in section 2.6, and analyzed the transcriptome using RNA-seq. Principal component analysis (PCA) was used to evaluate intergroup differences and intragroup sample duplications. We performed PCA on the PCC matrix of the gene expression values (FPKM) of all samples, as shown in **Figure 7A**. The samples between the groups were dispersed into three areas. All three diabetic mice clustered together in the bottom-left quadrant (PC1 < 0.0, PC2 < 0.0) and all three Ctl-Treg-treated mice clustered in the top-left quadrant (PC1 < 0.0, PC2 > 0.0). Two of the three nontreated mice clustered with the Ctl-Treg mice in the top left quadrant (PC1 < 0.0 and PC2 > 0.0). Most importantly, two of the three InsB:R3-Treg treated mice clustered in the top right quadrant (PC1 > 0.0 and PC2 > 0.0), which was distinct from the other groups. One InsB:R3-Treg treated mouse fell outside the top right quadrant and clustered in the top left quadrant with the Ctl-Treg and nontreated mice. Notably, this outlier had the lowest unique mapping rate of 64.61% (**Supplemental Table 1**). Therefore, PCA revealed that InsB:R3-Treg treated mice had the most different features compared with diabetic mice (**Figure 7A**), with Ctl-Treg and nontreated mice localized in the quadrants between the diabetic and InsB:R3-Treg treated mice.

The number of differential genes (including upregulation and downregulation) for each comparison combination and individual mouse is shown in a histogram (**Figure 7B** and **Supplemental-Figure 4A**). All the differentially expressed genes in the comparison group were pooled into differential gene sets. We used mainstream hierarchical clustering to cluster the FPKM values of the genes and homogenize the rows (Z-score). **Figure 7B** showed the three diabetic mice (pink) gathered together, and most of the Ctl-Treg and Non-treated mice gathered adjacent to the diabetic mice. However, two InsB:R3-Tregs were away from diabetic mice. **Supplemental Figures 4B–H** shows the upregulated and downregulated genes in the InsB:R3-Treg group relative to those in the other three groups. Notably, InsB:R3-Treg mice exhibited a significantly upregulated expression of insulin secretion-related genes. Our data suggest that InsB:R3-Treg treated mice maintain normal beta cell function.

To investigate the effect of InsB:R3-Tregs on islet inflammation, the FPKM value was used to evaluate the expression of autoimmunity-related genes (**Figures 7C–H**). Compared to the DM group, the InsB:R3-Treg and Ctl-Treg groups showed lower expression levels of Cd19, Cd4, Pdcd1, Tnfrsf18, and Stat6 and higher expression of Tnfrsf9. As B cells and CD4 T cells are the main effector cells of islet autoimmunity and PD-1 is upregulated in activated T cells, our data showed that Tregs downregulated both B cells and CD4 T cells and their activation status. Furthermore, the InsB:R3-Treg treated mice showed greater variance than the other treatment groups. For example, some mice showed extremely low levels of Cd19, Cd4, Pdcd1, and Tnfrsf18, whereas others showed gene expression levels similar to those in Ctl-Treg mice. To analyze the impact of InsB:R3-specificity on Tregs, we determined the gene expression between InsB:R3-Treg and Ctl-Treg group using Log2 fold changes. **Figure 7I** showed the InsB:R3-Treg group had reduced expression of B and T cell feature genes (Cd19, Cd4, and Cd8) and cell activation-related genes (Ctla4, Pdcd1, Cd80, Cd86, and Cd69), but increased expression of insulin-related genes (Pdx1 and Ins1). In summary, our data show that InsB:R3-Tregs influence the relative quantity of gene expression in B and T cells and T-cell activation-related genes, although the extent of the changes in individual mice varies.

### 3.6 InsB:R3-Tregs reduce the severity of insulitis and preserve the islet without causing systemic immune suppression

**Figure 8:**
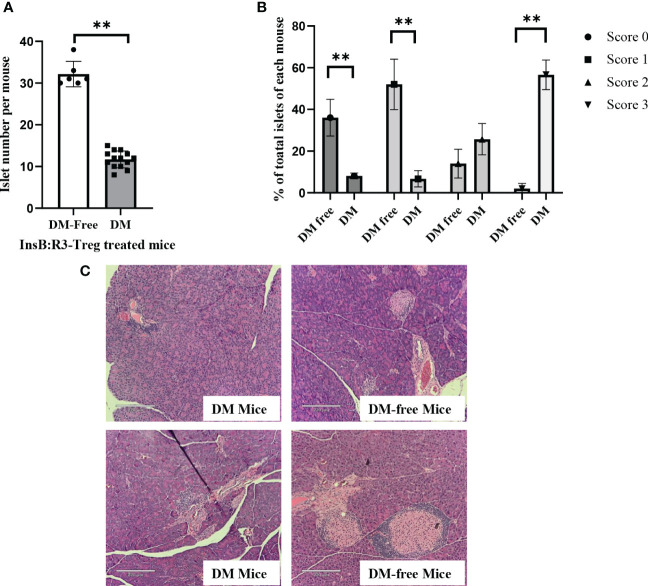
The islet preservation using InsB:R3-Treg treatment. **(A)** The islet numbers of the diabetes-protected mice (DM-Free, n=6) and diabetic mice (DM, n=14) treated with InsB:R3-Tregs. **(B)** Insulitis scores of DM-Free (n=6) and DM (n=14) mice. Insulitis score criteria was described in section 2.8. **p<0.001 was obtained using non-paired Student’s t test. **(C)** Representative pancreas hematoxylin and eosin staining from two DM-Free mice and two diabetic mice.

To evaluate the long-term benefits of InsB:R3-Tregs in preserving islets, we collected pancreata from six diabetes-protected mice (DM-Free) at 20 weeks and six diabetic mice following diagnosis via H&E staining. We then quantified the number of islets and evaluated the insulitis scores for each mouse. Compared with diabetic mice, DM-free mice had a significantly higher number of islets that were mainly intact or had mild insulitis (**Figures 8A, B**). In contrast, all diabetic mice had severe insulitis, and mild insulitis or intact islets were rarely observed. **Figure 8C** shows representative islets from the DM-Free and diabetic mice. The histological differences between the pancreatic islets of diabetes-free InsB:R3-Treg treated mice and diabetic control mice indicated that islet destruction was markedly reduced in the pancreas of mice treated with InsB:R3-Tregs.

**Figure 9:**
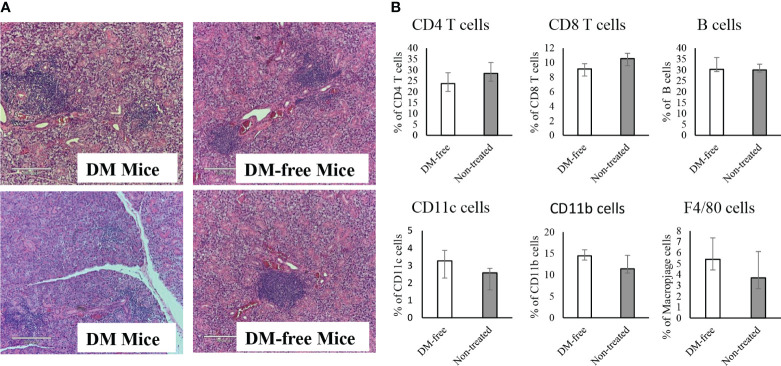
Evaluation of sialitis and sub-population of immune cells in the InsB:R3-Treg treated mice. **(A)** Salivary glands from DM-Free mice and DM mice treated with InsB:R3-Tregs were stained with hematoxylin and eosin. Representative images are shown. **(B)** The diabetes protected NOD.CD28^-/-^ mice (n=6) by InsB:R3-Tregs were euthanized at 20 weeks and splenocytes were stained with indicated antibodies and analyzed by flowcytometry. Eight weeks old NOD.CD28^-/-^ mice were used as control as the non-treated mice all developed diabetes by age of 13 weeks. The percentages of splenic CD4^+^ T cells, CD8^+^ T cells, CD19^+^ B cells (by antibody staining), CD11c^+^ Dendritic cells, and F4/80^+^ Macrophages cells were plotted. Cells were gated on DAPI negative lymphocytes. No significant differences were observed between two groups using unpaired Student’s t test.

Similar to NOD mice, NOD.CD28^-/-^ mice also develop sialitis. To investigate whether InsB:R3-Treg treatment has off-target effects, we assessed the outcomes of sialitis. We evaluated the development of sialitis in InsB:R3-Treg treated diabetes-free mice and diabetic mice from the saline and Ctl-Treg groups. **Figure 9A** shows that diabetes-free mice treated with InsB:R3-Treg developed sialitis, similar to control group mice. These data suggest that InsB:R3-Treg therapy does not suppress salivary gland inflammation, indicating that immune reactivity outside the pancreas is not suppressed.

To determine whether the infusion of InsB:R3-Tregs had any systemic side effects on the immune system, we evaluated the populations of CD4^+^ and CD8^+^ T cells, B cells, macrophages, and dendritic cells in the spleens of DM-Free mice and non-treated NOD.CD28^-/-^ mice. Compared to non-treated mice, DM-Free mice treated with InsB:R3-Treg did not show any significant changes in immune components (**Figure 9B**). These data suggested that InsB:R3-Treg therapy did not cause detectable systemic changes in the immune system.

## 4 Discussion

Previous studies using vaccines, monoclonal antibodies, and CAR-CD8 T cells targeting the pathogenic MHCII/Insulin B:9-23 register 3 conformational epitope paved the foundation for CAR-Treg studies (7, 19, 20, 23–25). This study showed that engineered IA^g7^/InsB:R3-targeting Tregs could significantly protect mice against diabetes in a non-preconditioned spontaneous T1D mouse model. InsB:R3-CARs confer islet antigen specificity on polyclonal Tregs. Upon engagement with MHC II/InsB:R3, the engineered CAR-Tregs proliferated, secreted protective cytokines, and inhibited the co-culture of diabetic polyclonal effector T-cells. *In vivo*, InsB:R3-Tregs tend to be enriched in draining lymph nodes, where relatively more antigens can be found, than in non-draining lymph nodes. Second-generation InsB:R3-CAR with antigen affinity at the nanomolar level enables engineered Tregs to protect against T1D. To the best of our knowledge, this is the first successful application of a TCR-like CAR specific for MHC II/InsulinB:9-23 register 3 epitope to guide regulatory T cells in inhibiting autoimmune diabetes.

This study demonstrates that the MHCII/Insulin B:9-23 epitope with the peptide presented in a pathogenic position is a key target in developing islet-antigen-specific Tregs to treat T1D. Previous studies by our group and others have shown that presentation of the insulin B:9-23 peptide in register 3 by IA^g7^ is critical for activating a key population of pathogenic T cells and the concurrent initiation of islet autoimmunity in NOD mice (7, 19, 20, 23–25, 58). Another report showed that IA^g7^/InsB:R3 reactive naive CD4^+^ T cells become effector T cells when mice age and develop diabetes (58). MAb757, the parent antibody of InsB:R3-CAR, has high binding specificity to IA^g7^/InsB:R3 antigens but has no cross-reactivity with insulin B:9-23 or the peptide presented in other registers. In contrast, insulin-specific Tregs did not have a protective effect against T1D. Hardtke-Wolenski et al. generated CAR-Tregs using an antibody specific for insulin, but not for the MHCII/insulin antigen (59). Insulin-CAR-Tregs were unable to prevent spontaneous diabetes in NOD mice, although the cells survived long term in diabetic mice (59). Therefore, this and our previous studies confirmed that the MHCII/Insulin conformational epitope is a valid target for T1D.

After antigen spread, the efficacy of the immune intervention largely relies on its capacity to suppress polyclonal lymphocytes. Our data showed that expanded and activated InsB:R3-Tregs could suppress the proliferation of nearby CD4 and CD8 effector T cells, regardless of their antigen specificities. Consistent with InsB:R3-Treg suppression *in vitro*, we observed that the administration of InsB:R3-Tregs significantly reduced the severity of insulitis, preserved functional islets, and consequently prevented the onset of disease in a dose-dependent manner. Consistent with the observed disease prevention, InsB:R3-Treg cells survived in PLNs and islets. Compared with the Ctl-Treg group, InsB:R3-Tregs reduced the gene expression of effector T cells, B cells, and inflammatory cell activation molecules, suggesting that InsB:R3-Tregs reduce islet inflammation. Following one infusion at the pre-diabetic stage, InsB:R3-Tregs were detected in the pancreatic lymph nodes and islets after one week; however, the number of InsB:R3-Tregs found in the diabetes-protected mice at 20 weeks was insignificant. Undetectable InsB:R3-Tregs may be related to alleviated islet destruction, resulting in reduced release of islet antigens. We previously reported that InsB:R3-CD8 T cells temporarily delayed the onset of T1D (25). InsB:R3-Treg therapy, which postpones disease up to two healthy lifespans, is superior to the use of engineered CD8^+^ T cells. Compared to the parental antibody Ab757, which requires weekly injections to modulate diabetes (23, 24), InsB:R3-Treg therapy is a superior therapeutic modality, as only one dose of Tregs successfully delayed T1D.

Compared to polyclonal Treg cells, antigen-specific wild-type Treg cells are more effective in preventing autoimmune disease (41–44). We found that two million InsB:R3-Tregs significantly protected mice against disease, whereas the same number of non-islet antigen-specific polyclonal Tregs did not show any protective effect. Our data are consistent with those of previous studies on wild-type BDC2.5 Tregs targeting the peptide of chromogranin A (ChgA) (41). In NOD mice, two million BDC2.5 Tregs are required to block the ability of twenty-five million diabetogenic NOD spleens and LN cells to transfer disease in immune-deficient mice. In contrast, five million polyclonal Tregs showed no effect. Compared to two million InsB:R3-Tregs, one million cells only slightly postponed the onset of diabetes, whereas half a million cells did not show any protective effect. Our findings suggest that both islet antigen specificity and cell quantity are essential for Tregs to home to islet lesions and effectively inhibit islet inflammation.

BDC2.5 Tregs, targeting the ChgA antigen, showed the strongest disease protection in all treated mice (41). Two million InsB:R3-Tregs or BDC2.5 Tregs are required to prevent NOD. CD28^-/-^ mice from diabetes (41). Compared to the complete disease protection of BDC2.5 Tregs, InsB:R3-Tregs only prevented half of the treated mice from disease and postponed onset of T2D in the rest mice. Multiple reasons may explain the different efficacies of these two types of Tregs. First, BDC2.5 Tregs are primary cells from transgenic mice, whereas InsB:R3-Tregs are generated via virus transduction. Therefore, BDC2.5 Tregs were healthier than the manipulated Tregs. Second, antigen abundance and strength affect Treg activation and function (60, 61). The survival of T-cells depends on antigen stimulation. According to MHC II/peptide tetramer staining, NOD mice have significantly more IA^g7^/ChgA positive T cells than IA^g7^/Insulin positive T cells, suggesting that ChgA antigens are more abundant than InsB:R3 in islets (23, 62). The binding of the B:9-23 peptide in register 3 is highly unfavorable, and the B:9-23 peptides are likely occupied predominantly by non-disease-related high-affinity registers (7, 19). Therefore, upon transfer, InsB:R3-Tregs may not survive as long as BDC2.5-Tregs because of limited antigens.

In this and our previous studies, we used NOD.CD28^-/-^ mouse model to evaluate the efficacy of Treg cell therapy because of the consistency and robustness the model offers. The standard NOD mice, widely used in the field, have variable incidence of diabetes depending on the colony location, sex, housing conditions, diet, and experimental handling (63–66). The model’s sensitivity to environmental factors and experimental procedures can introduce known and unknown biases in the data. On the other hand, the NOD.CD28^-/-^ mice do not suffer from any of the issues listed above. All the mice reliably develop insulitis between 3-4 weeks of age, all progress to severe insulitis by 6 weeks of age, followed by progressive and synchronous decrease of insulin mRNA in islets, leading to overt diabetes by 10-14 weeks of age in ~100% mice (67), regardless of colony locations (UChicago, UCSF, Stanford, Baylor, and Indiana Univ), sex, cage, bedding, water, diet, and enrichment. This makes the NOD.CD28^-/-^ mouse model reliable and sensitive in detecting changes in diabetes development and progression. Data generated in this model can be more confidently attributed to the experimental conditions, not unknown and uncontrolled alteration of the mice or the environment. Another reason that NOD.CD28^-/-^ is our preferred model for evaluating Treg therapy is that the mice are relative Treg deficient. By Foxp3 staining, they have 50% reduction of Tregs and by CD25 staining, they have about 80% reduction (68). This makes these mice good recipients for Treg “add back” experiments in our study.

The NOD.CD28^-/-^ have a few other distinctions that are worth pointing out. First of all, the pathology in the islets has a CD8 dominance when compared to the standard NOD (67). This is likely due to the relative CD28 independence of CD8 effector T cells when compared to CD4 effector T cells. This feature is not necessarily a drawback. In fact, nPOD studies of islets from human T1D found CD8 to be more frequent than CD4 T cells (69, 70). Moreover, the NOD.CD28^-/-^ mice are relatively deficient in IL-2 (68). This can impair the survival of infused Tregs and may also impede the generating of new Tregs important for infectious tolerance. Lastly, the effector T cells in the NOD.CD28^-/-^ mice emerged with relatively weak opposition of Treg-mediated suppression thus they are likely more sensitive to suppression by infused Tregs than effectors in the standard NOD mice. Taking together, we believe that NOD.CD28^-/-^ mice offer one dependable platform for *in vivo* evaluation of Treg cell therapy for T1D. It can complement other models such as adoptive transfer of NOD diabetogenic T cells in T cell deficient recipients (as we have done), and prevention and diabetes reversal in standard NOD mice.

While we focused on disease prevention by InsB:R3-Tregs in this study, we preliminarily examined intra-islet immune changes following treatment. RNA sequencing and flow cytometry analyses indicated that intra-islet effector lymphocytes showed varied responses to the treatment. This was consistent with the partial disease protection provided by InsB:R3-Tregs. Currently, the mechanism via how Tregs migrate toward islets and why only some mice are protected remain unclear. Further studies should be conducted to explore the potential mechanisms underlying the protective effects of InsB:R3-Tregs and to enhance the efficacy of Treg disease protection.

In summary, we demonstrated for the first time that engineered regulatory T cells targeting the pathogenic MHCII/insulin epitope with the insulin B:9-23 peptide trapped in a disease-related register could effectively and safely protect NOD.CD28^-/-^ mice against T1D. To achieve high disease protection efficacy, islet antigen specificity, a sufficient quantity of cells, and CARs with relatively high affinity are required for MHCII/insulin-specific CAR-Tregs. The present study strongly supports the use of engineered regulatory T cells that target disease-relevant antigens to inhibit islet autoimmunity and prevent T1D. This therapy is relatively safe, with no detectable off-target systemic side effects. Currently, no effective and safe antigen-specific immunological therapies are available for treating autoimmune diabetes in humans. Given the great similarity in the pathogenesis of T1D between humans and NOD mice, our group is actively exploring immunotherapies that target human leukocyte antigen/insulin antigens.

## 5 Nomenclature

APC, antigen presenting cell; B:9-23, insulin B chain amino acid 9 to 23; BFP, blue fluorescent protein; CAR, chimeric antigen receptor; CD, cluster of differentiation; DM, diabetes; DM-Free, diabetes-free; HEL, Hen Egg Lysozyme; Ctl-CAR, control-CAR; DEG, differentially expressed genes; DGE, differential gene expression; ELISA enzyme-linked immunosorbent assay; FoxP3, forkhead box protein P3; FPKM, fragments per kilobase of transcript per million mapped reads; G2-CAR, the second generation chimeric antigen receptor; GO, Gene Ontology; HEL, hen egg-white lysozyme; ILN, inguinal lymph nodes; InsB:R3, insulin B:9-23 peptide presented in register 3; InsB:R3-CAR, InsB:R3-targeting CAR; KEGG, Kyoto Encyclcopedia of Genes and Genomes; MFI, Mean Fluorescence Intensities; MHC, major histocompatibility complex; MHC II, major histocompatibility complex class II; Min, minutes; NOD mice, non-obese diabetes mice; Non-T, non-Treg treated; NSG mouse, NOD scid gamma mouse; PCA, principal component analysis; PCC, the Pearson correlation coefficient; PLN, pancreatic lymph node; R3, register 3; T1D, type 1 diabetes; TCR, T cell receptor; Teff, effector T cell; Tetr, tetramer; Treg, regulatory T cell.

## Data availability statement

The data presented in the study are deposited in the ArrayExpress Gene Expression Omnibus (GEO) repository, accession number GSE238146.

## Ethics statement

The animal study was reviewed and approved by Indiana University School of Medicine Institutional Animal Care and Use Committee (IACUC).

## Author contributions

NO, DP, RB, NA, JC, DE and SR performed the experiments. LZ wrote and edited the manuscript. KY, TJ and QT edited the manuscript. All the authors contributed to the article and approved the submitted version. LZ is the guarantor of the data.
