# Corrigendum: Regulatory T cells targeting a pathogenic MHC class II: insulin peptide epitope postpone spontaneous autoimmune diabetes

**DOI:** 10.3389/fimmu.2024.1391518

**Published:** 2024-03-07

**Authors:** Nyerhovwo Obarorakpor, Deep Patel, Reni Boyarov, Nansalmaa Amarsaikhan, Joseph Ray Cepeda, Doreen Eastes, Sylvia Robertson, Travis Johnson, Kai Yang, Qizhi Tang, Li Zhang

**Affiliations:** ^1^ Diabetes Center, Indiana Biosciences Research Institute, Indianapolis, IN, United States; ^2^ Department of Medicine, Endocrinology, Diabetes & Metabolism, Baylor College of Medicine, Houston, TX, United States; ^3^ Department of Biostatistics and Health Data Science, School of Medicine, Indiana University, Indianapolis, IN, United States; ^4^ Melvin and Bren Simon Comprehensive Cancer Center, Experimental and Developmental Therapeutics, School of Medicine, Indiana University, Indianapolis, IN, United States; ^5^ Center for Computational Biology and Bioinformatics, School of Medicine, Indiana University, Indianapolis, IN, United States; ^6^ Herman B Wells Center for Pediatric Research and Department of Pediatrics, Indiana University School of Medicine, Indianapolis, IN, United States; ^7^ School of Medicine, Indiana University Bloomington, Bloomington, IN, United States; ^8^ Diabetes Center, University of California San Francisco, San Francisco, CA, United States; ^9^ Department of Surgery, University of California San Francisco, San Francisco, CA, United States; ^10^ Gladstone Institute of Genomic Immunology, University of California San Francisco, San Francisco, CA, United States; ^11^ Center for Diabetes and Metabolic Diseases, Indiana University School of Medicine, Indianapolis, IN, United States; ^12^ Biochemistry & Molecular Biology, Indiana University School of Medicine, Indianapolis, IN, United States

**Keywords:** Type 1 diabetes, regulatory T cell, chimeric antigen receptor, antigen specific immunotherapy, MHC II/Insulin complex, monoclonal antibody

In the published article, there was an error in the **Funding** statement. Grant “JDRF 2-SRA-2018-648-S-B” grant was missing in the statement. The correct **Funding** statement appears below.

**Funding**

This study was supported by grants from NIH R03AI139811-01A1, DoD W81XWH2210087, JDRF 2-SRA-2018-648-S-B, and a Pilot and Feasibility Award from the CDMD NIH/NIDDK Grant Number P30 DK097512 (to LZ).

The authors apologize for this error and state that this does not change the scientific conclusions of the article in any way. The original article has been updated.

