# Supplementary material for: Regulatory T cells targeting a pathogenic MHC class II: Insulin peptide epitope postpone spontaneous autoimmune diabetes
Source: Front Immunol. 2023 Aug 1;14:1207108. doi: 10.3389/fimmu.2023.1207108 (PMC10428008; doi:10.3389/fimmu.2023.1207108)
Supplement: Supplementary file 1 [file DataSheet_1.docx]

Supplementary Material

Regulatory T cells targeting a pathogenic MHC class II: Insulin peptide epitope postpone spontaneous autoimmune diabetes

Nyerhovwo Obarorakpor ^1^, Deep Patel ^1^, Reni Boyarov ^1^, Nansalmaa Amarsaikhan ^1^, Joseph Ray Cepeda ^2^, Doreen Eastes ^1^, Sylvia Robertson ^1^, Travis Johnson ^1,3,4,5^, Kai Yang ^6,7^, Qizhi Tang ^8,9,10^, Li Zhang ^1,11,12 *^

^1^ Diabetes Center, Indiana Biosciences Research Institute, Indianapolis, IN, United States, ^2^ Department of Medicine, Endocrinology, Diabetes & Metabolism, Baylor College of Medicine, Houston, TX, United States, ^3^ Department of Biostatistics and Health Data Science, ^4^ Melvin and Bren Simon Comprehensive Cancer Center, Experimental and Developmental Therapeutics, ^5^ Center for Computational Biology and Bioinformatics, School of Medicine, Indiana University, Indianapolis, IN, United States, ^6^ Herman B Wells Center for Pediatric Research and Department of Pediatrics, Indiana University School of Medicine, Indianapolis, IN, United States,^7^ School of Medicine, Indiana University Bloomington, Bloomington, IN, United States, ^8^ Diabetes Center, ^9^ Department of Surgery, ^10^ Gladstone Institute of Genomic Immunology, University of California San Francisco, San Francisco, CA, United States, ^11^ Center for Diabetes and Metabolic Diseases, Indiana University School of Medicine, Indianapolis, IN, United States, ^12^ Biochemistry & Molecular Biology, Indiana University School of Medicine, Indianapolis, IN United States.

*Corresponding author: Li Zhang: LZhang@Indianabiosciences.org

Supplemental Table 1: Comparison of sample and reference genomes

| **sample** | **total reads** | **total map** | **unique map** | **multi_map** |
| --- | --- | --- | --- | --- |
| DM1 | 49032242 | 46319686(94.47%) | 40037127(81.65%) | 6282559(12.81%) |
| DM2 | 43594164 | 40924173(93.88%) | 35357041(81.1%) | 5567132(12.77%) |
| DM3 | 51095970 | 48233620(94.4%) | 42472459(83.12%) | 5761161(11.28%) |
| NonT1 | 41934478 | 40040819(95.48%) | 29411929(70.14%) | 10628890(25.35%) |
| NonT2 | 40618994 | 38568502(94.95%) | 30694926(75.57%) | 7873576(19.38%) |
| NonT3 | 33183666 | 31735509(95.64%) | 28325127(85.36%) | 3410382(10.28%) |
| InsT1 | 39267972 | 37498813(95.49%) | 35438759(90.25%) | 2060054(5.25%) |
| InsT2 | 50830176 | 48923814(96.25%) | 46190686(90.87%) | 2733128(5.38%) |
| InsT3 | 49716610 | 47629148(95.8%) | 32122032(64.61%) | 15507116(31.19%) |
| CtlT1 | 159901484 | 152723967(95.51%) | 123391607(77.17%) | 29332360(18.34%) |
| CtlT2 | 51519560 | 46464067(90.19%) | 33460318(64.95%) | 13003749(25.24%) |
| CtlT3 | 71533384 | 64158995(89.69%) | 54300714(75.91%) | 9858281(13.78%) |

Supplemental Figure 1


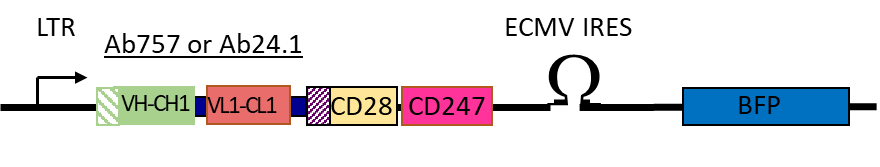


S-Figure 1: Cartoon illustrating the main features of the 2^nd^ generation 2 (G2) retroviral CAR construct. CARs encode the antibody light chains (green boxes) including their native leader peptides (shaded box) joined by a flexible linker to the VH and CH1 domains (orange boxes) and fused to the transmembrane (shaded) and signaling domains of CD28 (yellow boxes) and cytosolic domain of CD247 [CD3ζ] (red boxes). The construct also contains an IRES driven BFP to allow detection of transduced cells. Expression is driven by the viral LTR.

Supplemental Figure 2


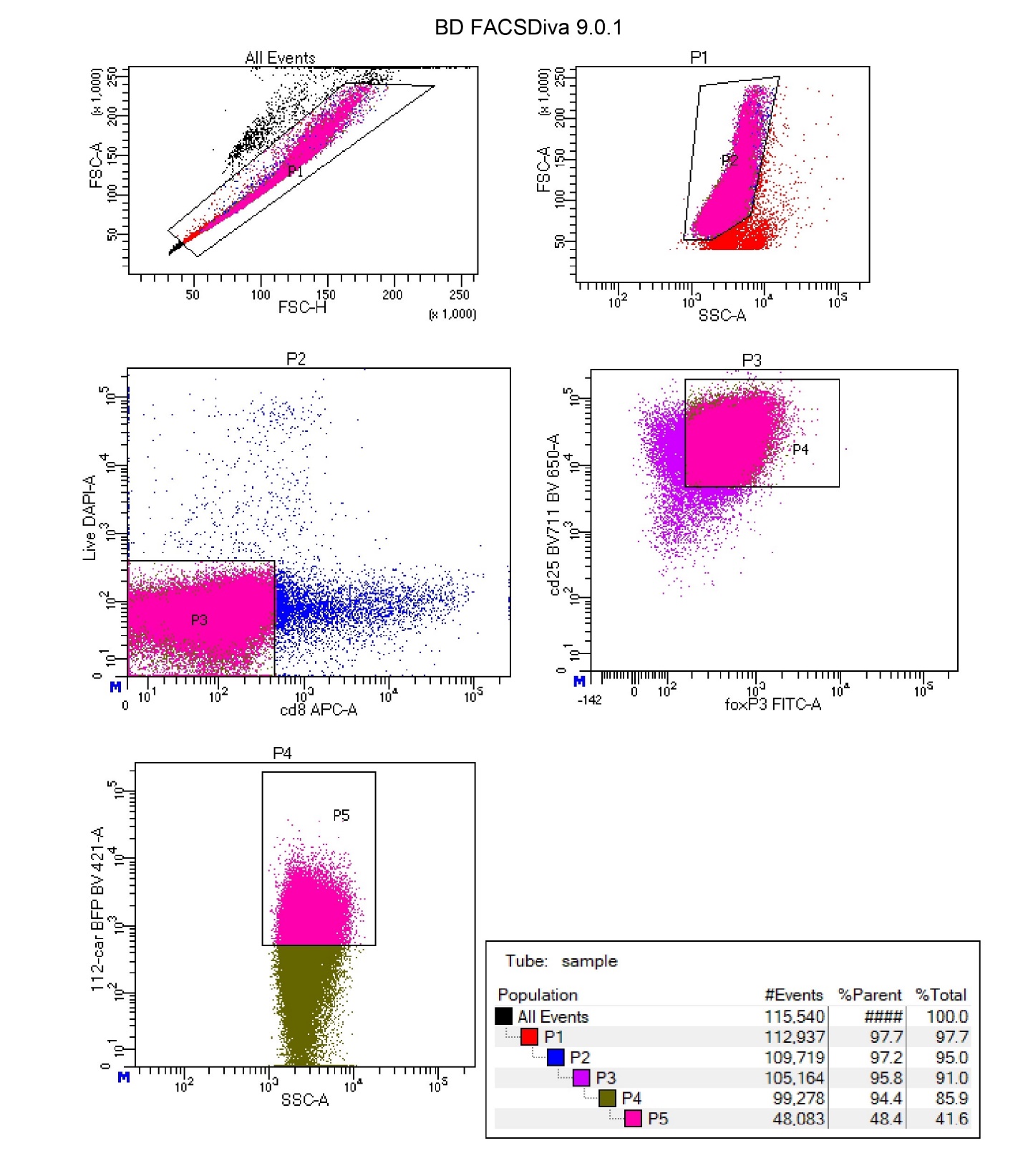


S-Figure 2: Representative CAR-Treg sorting strategy. The CD4^+^CD25^Hi^CD62L^+^CD8^-^FoxP3^GFP+^ Treg cells were transduced with retrovirus encoding CAR-BFP. After expansion, the CARTregs were sorted by gating on alive CD8^–^ CD25^Hi^ Foxp3^GFP+^CAR^BFP+^ cells. Cells were sorted with BD Sorp Aria sorter.

Supplemental Figure 3:


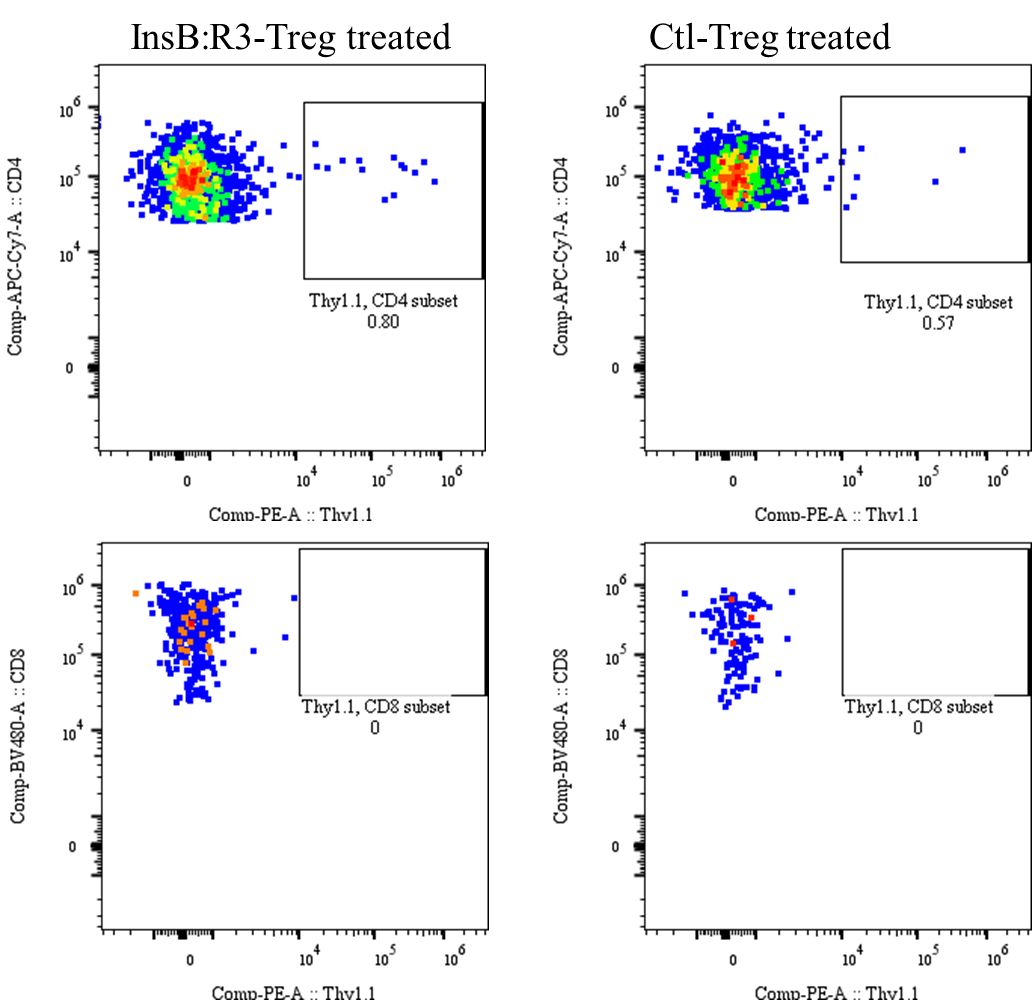


S-Figure 3: The adoptive transferred Thy1.1 Tregs were detected in islets of NOD.CD28^-/-^ mice on day 8 post transfer. CARTregs were generated from Thy1.1FoxP3^GFP^ mice and transferred to NOD.CD28^-/-^ mice. On day 8 post-transfer, islets were hand-picked and pooled together, two mice per group, and cultured overnight. The islet infiltrating lymphocytes were collected and stained with CD4, CD8 and Thy1.1 antibodies. Percentages of Thy1.1^+^ Tregs of CD4 T cells per pancreas were plotted. CD8 T cells were used as controls as the transferred Tregs are CD8 negative. The experiment was repeated with similar results.


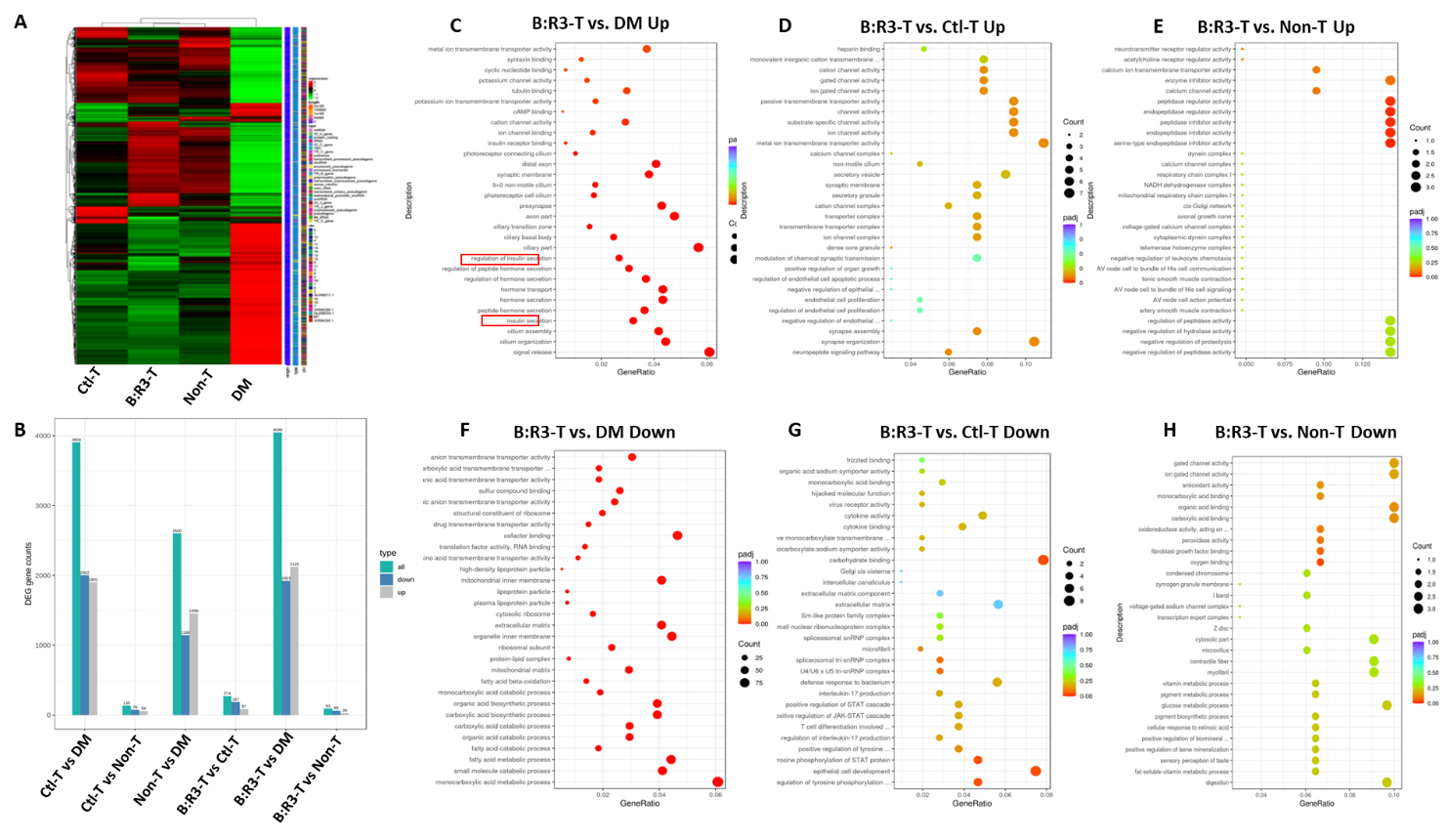


S-Figure 4: Islet RNAseq analysis. **(A)** The heat map of the FPKM value of differential expression genes in groups. **(B)** Difference Comparison Combine Differential Gene Number Statistics Histogram. Blue and gray represent the differential genes for down-regulation and up-regulation, respectively, and the numbers on the columns indicate the number of differential genes. **(C-H)** Gene Ontology (GO) Enrichment Analysis Scatter Plot. The abscissa in the graph is the ratio of the differential gene number to the total number of differential genes on the GO Term, and the ordinate is GO Term. padj: The corrected p value of multiple hypothesis test. GO terms with padj < 0.05 are significant enrichment.
